# Transient Carbon Reserves in Barley: Malate, Sucrose and Starch Are the Main Players, Their Quantitative Involvement Being Light Intensity Dependant

**DOI:** 10.3389/fpls.2020.00209

**Published:** 2020-03-06

**Authors:** Kallyne A. Barros, Alberto A. Esteves-Ferreira, Masami Inaba, Helena Meally, John Finnan, Susanne Barth, Ronan Sulpice

**Affiliations:** ^1^Plant Systems Biology, School of Natural Sciences, Ryan Institute, National University of Ireland, Galway, Ireland; ^2^Teagasc, Crops, Environment and Land Use Programme, Crop Science Department, Carlow, Ireland

**Keywords:** barley, growth, diurnal metabolism, transient reserves, sucrose, malate, starch, *Hordeum vulgare*

## Abstract

Under natural environment plants experience different light intensities which can affect photosynthesis and consequently the availability of carbohydrates for daytime growth and their transient storage to supply night growth. We grew a spring barley cultivar, Propino, under three different light intensities under warm days and nights, and evaluated the spatial and diurnal adjustments occurring in the transient carbon stores. Leaves under high light at the end of the day accumulated mainly sucrose (30%) and malate (35%), with lower content of hexoses (5%), starch (15%) and fructans (15%). Under low light, plants presented reduced photosynthesis, with lower metabolite contents at end of day. The malate represented 51% of the total carbon accumulated at end of the day, at the expense of sucrose (12%), other metabolite contributions remaining similar to high light. The percentage of metabolites consumed at night was similar for all light intensities with around 75% of the sucrose and starch being mobilized whilst malate and fructans were only partially mobilized with 56 and 44%, respectively. Altogether, sucrose and malate were the main contributors of the total carbon used at night by barley plants, sucrose being predominant under high light (35% vs. 27%), but malate being the major metabolite used under low light with 40% of the total carbon consumed. Interestingly, light intensity also influenced the location of the C transient stores, the plants under low light prioritizing the accumulation of the metabolites, mostly malate, in the youngest tissues. Therefore, light influences quantitatively, but also qualitatively and spatially the carbon stores in the spring barley cv. Propino, suggesting a tight regulation of the primary metabolism.

## Introduction

Plants grown in the natural environment experience variation in both light quality and quantity. In response, plants need to adjust their growth and metabolism to cope with the changes (Annunziata et al., 2017). The development of leaf primordia and flowering responses in barley are very sensitive to light intensity, quality and day length. In barley, leaf development and flowering time are faster under higher light intensities and longer days, requiring far-red light as well (Aspinall and Paleg, 1963; Paleg and Aspinall, 1964). Light intensity and photoperiod can directly affect the light harvesting complexes and lead to changes in the abundance of chlorophyll and fluorescence parameters (Bailey et al., 2001; Kouřil et al., 2013). Light intensity effects in barley include changes in chlorophyll *a*/*b* ratio within light-harvesting complexes, the levels of Q_b_ and Cyt*f* and thus electron transport rate (De la Torre and Burkey, 1990). Also, in Arabidopsis, the activation state of Rubisco activase, which is controlled by the redox state of the cell, is responsive to light intensity, and the proper regulation of the activity of Rubisco activase is crucial to acclimation to light fluctuation and optimal growth (Carmo-Silva and Salvucci, 2013).

Plants grow during both day and night although the assimilation of carbon (C) resources occurs only in the presence of light and mostly in mature leaf tissues, demanding a spatial and temporal control of the partitioning and use of C (Walter et al., 2009). Photoassimilates are stored in both source and sink tissues, not solely for daily night requirements for growth and maintenance, but also over long time for supplying flowering and grain development (Smouter and Simpson, 1991; Schnyder, 1993; Wang and Tillberg, 1996).

The fate of newly assimilated C in barley has been described to be primarily incorporated in sucrose, then starch and other C forms (Gordon et al., 1979; Gordon et al., 1982). However, the mechanisms regulating the partitioning and usage of photoassimilates are still not clear. To get a better understanding of the mechanisms involved, it is necessary to analyze photoassimilates in sink and source tissues separately, as well as document their diurnal use.

The partitioning and accumulation of photoassimilates can be controlled by environmental factors, by developmental signals, and the circadian clock. Hormone signaling and the circadian clock coordinate growth and the metabolism of transient reserves in Arabidopsis. It has been hypothesized that the clock has a lesser role in monocots, based on their linear diurnal growth patterns and the strong impact of environmental factors on growth (Covington et al., 2008; Walter et al., 2009; Poire et al., 2010; Feugier and Satake, 2013, 2014). The influence of environmental factors on the C reserves has been extensively studied in both monocots and dicots, e.g. temperature (Lorenzo et al., 2015), light intensity (Nagaraj et al., 2001), photoperiod (Pokhilko et al., 2014) and nitrogen status (Wang and Tillberg, 1996; Wang et al., 2000; Lattanzi et al., 2012). However, the diurnal regulation and allocation between mature and growing tissues of the C reserves has been poorly described in monocots. In particular little is known about the qualitative and quantitative adjustments occurring in response to light intensity.

In this work, we investigate in the spring barley cultivar Propino the role of light intensity in the regulation of diurnal growth and C metabolism. For this purpose, we estimated CO_2_ assimilation, and determined the diurnal variations of the C stores in all above ground organs of young barley plants grown under three contrasting light intensities.

## Materials and Methods

### Plant Material, Growth Conditions and Harvesting

Barley seeds (*Hordeum vulgare* L.) of the spring cultivar Propino were germinated in the dark at 24°C for 3 days on dampened paper and then transferred to a growth chamber equipped with LED lights (C75-NS1, C75-AP67, Valoya Oy, Helsinki, Finland) into pots with Bord na Móna potting substrate plus^+^ (Bord na Móna Horticulture Ltd., Ireland), one seed per pot.

Propino plants were grown under three photosynthetically active radiation (PAR) intensities: 100 μmol photons m^–2^ (LL), 300 μmol photons m^–2^ s^–1^ (ML) and 500 μmol photons m^–2^ s^–1^ (HL). The photoperiod for all conditions was 16 h:8 h light:dark and the temperature was 22°C:18°C day:night. Plants were harvested when they reached 3 leaf stage, with the third leaf – youngest leaf – being 3–5 cm exposed. Sheaths and blades of each leaf were harvested separately. Three replicates were harvested at five timepoints covering a period of 24 h, each replicate consisting of three sheaths or blades from three different plants. Samples were frozen in liquid nitrogen, grinded to fine powder using a ball mill and stored at −80°C to further metabolic analysis.

### Elongation Rate and Fluorescence Parameters

Second and third blades were marked at the base of the blade at end of day (0 h), then at end of night period (8 h), and lastly at end of day (24 h). The elongation rates were calculated by measuring the distance between each mark and dividing by the number of hours of each period: 8 h for the night and 16 h for the daytime.

Chlorophyll *a* fluorescence parameters were determined using a PAM-2500 (Heinz Walz GmbH, Germany) on the second blade. The maximum photochemical quantum yield of PSII (*F*_v_/*F*_m_) and the effective photochemical quantum yield of PSII [Y(II)] were determined at steady state of chlorophyll *a* fluorescence with a saturation pulse of 8.000 μmol m^–2^s^–1^ (Pollock, 1986; Genty et al., 1989). ETR was calculated according to PAM-2500 handbook guidelines.

### Gas Exchange Measurements

The net photosynthesis (*A*_N_), the stomatal conductance (*g*_s_), sub-stomatal CO_2_ concentrations and transpiration (E) were measured in open system gas exchange (LI-6400XT, LI-COR, Lincoln, NE, United States, EUA). The temperature of the chamber was kept at 22°C for all measurements; the vapor pressure deficit (VPD) around 1.1 kPa, CO_2_ concentration at 400 ppm, light intensity of 500 μmol photons.m^–2^ s^–1^. The measurements were taken from the second leaf. *A*_N_, gs, and E were expressed on a FW basis. It was achieved by determination of the weight and surface of the second leaf.

### Water Content and Carbon Content Estimations

The water content was obtained from five disks of the second blade from six plants. The disks were excised and immediately weighed. Then the disks were dried in a drying cabinet at 70°C for 72 h and weighed again to obtain dry weight. The difference was used to calculate the percentage of water and dry matter per gram of fresh weight.

For the calculation of carbon accumulation and consumption, we used the metabolite content determined at end of day and end of night in the different plant organs, multiplying the concentration of metabolite by the number of carbon atoms present in each molecule, i.e. 6 for glucose, fructose, sucrose (equivalent glucose), fructans (equivalent glucose), starch (equivalent glucose) and 4 for malate. Then, C concentration at end of day (μmol C.g^–1^ FW) and C consumption at night (μmol C.g^–1^ FW) were estimated at whole plant levels by taking into account the respective weights of each organ per plant. The carbon consumption at night was estimated by the difference between the average content found at the first and fifth time points (ED and ED2) and the third time point (end of the night).

### Metabolite Analyses

For metabolite analyses, 20 mg of frozen powder was subjected to ethanolic extraction. Sequential extractions with ethanol concentrations of 98, 80, and 50% were performed and between each step the samples were incubated at 85°C for 20 min and the supernatants collected after centrifugation at 3220 *g* for 10 min (Cross et al., 2006). The ethanolic phase was used to determine fructans, sucrose, glucose, fructose and malate while starch and proteins were determined in the pellet. Glucose and fructose were determined according to Stitt et al. (1989) with minor modifications by using 0.6 U.μl^–1^ NAD^+^ dependant G6PDH. The determination of sucrose was performed using 0.25 U.μl^–1^ α-glucosidase (E-MALTS, Megazyme u. c., Ireland). The production of NADH was determined at 340 nm using a spectrophotometer model ELx800^TM^ (BioTek Instruments, Inc., United States).

Fructans were determined after completion of sucrose, glucose and fructose analyses, using the same determination plate. The NADH and enzymes used for sugar analyses present in the wells were denaturated by addition of 10 μl HCl 1 M and the plate was sealed and incubated at 95°C for 30 min. Then the plate was cooled on ice and extracts neutralized with 10 μl NaOH 1 M. To each well, 7 μl of acetate buffer 0.1 M pH 4.9 were added to the plate and 1 μl of a mix containing 0.1 U.μl^–1^. endo-inulinase and 0.1 U.μl^–1^ exo-inulinase (respectively, E-ENDOIAN, E-EXOIAN, Megazyme u. c., Ireland). The plate was then sealed and incubated overnight at 37°C. To determine fructans, 75 μl of Hepes buffer 0.5 M pH 7 containing 3 mM ATP and 1.3 mM NAD was added in each well. After obtention of a stable baseline at 340 nm, 1 μl of 0.6 U.μl^–1^ glucose-6-phosphate dehydrogenase, 1 μl 0.9 U.μl^–1^ hexokinase and 1 μl 0.3 U.μl^–1^ phosphoglucose isomerase were added sequentially for the determination of glucose and fructose molecules present in fructans. Starch was determined as previously described by Hendriks et al. (2003). Malate was determined according to Cross et al. (2006). Proteins were determined by the method described by Lowry et al. (1951), adapted to 96-well plate.

### Statistical Analyses

For growth and photosynthesis variables, ANOVA was applied with Tukey *post hoc* test using six replicates. For metabolites, ANOVA was applied with a Tukey *post hoc* test, using three replicates. All tests were conducted on IBM SPSS Statistics for Windows, Version 23.0. Armonk, NY, United States, IBM Corp. Means were considered significantly different at *P* < 0.05.

## Results

### The Impacts of Light Intensity on Growth, Fluorescence and Proteins

Plants grown under HL showed the fastest development, reaching three-leaf stage at 14 days after sowing (DAS), while plants grown under ML took 16 DAS and plants grown under LL 18 DAS. Plants grown under LL were the tallest but did not differ significantly in size from plants grown under HL, while plants grown under ML were the smallest, differing significantly from plants grown under LL (Supplementary Figure 1A). The total biomass of plants grown under LL was significantly lower than plants grown under ML and HL (Supplementary Figure 1B). The decrease of 80% in light intensity applied between HL and LL resulted in a delay of only 4 days to reach the third leaf stage.

At the time of harvesting, for the three treatments, the first leaf was mature and fully expanded while second and third blades were still growing. The elongation rates of second and third leaves were unaffected by light intensity and were similar during both daytime and night. The elongation rate of second leaves, which are reaching maturity, were lower than third leaves (Supplementary Figure 2). Although plants showed statistically significant differences for maximum photochemical quantum yield of photosystem II (*F*_v_/*F*_m_) and the effective photochemical quenching of photosystem II – Y(II), the changes observed were very small (Table 1). Thus, the electron transport rate (ETR) varied a lot as it is dependent on light intensity. LL plants showed a higher water content than HL plants, ML plants being intermediate. Photosynthesis was significantly reduced in plants under ML and LL compared to HL, while *g*_s_, C_i_, E showed higher values only under HL, leading to a much lower water use efficiency (WUE) in these plants (Table 2).

**TABLE 1:** Water content and fluorescence parameters of cv. Propino plants grown under different light intensities.

	Water content (%)	F_v_/F_m_	Y_(II)_	ETR
HL	88.0 ± 1.2 a	0.790 ± 0.005 b	0.743 ± 0.004 a	156.0 ± 0.9 c
ML	90.5 ± 0.8 ab	0.794 ± 0.004 b	0.767 ± 0.007 c	96.7 ± 0.8 b
LL	91.7 ± 3.5 b	0.769 ± 0.005 a	0.750 ± 0.004 b	31.5 ± 0.2 a

*Plants were grown in a 16 h:8 h light:dark photoperiod 22°C:18°C day:night under 500 μmol photons m**^–^**^2^s**^–^**^1^ for 14 DAS (HL), 300 μmol photons m**^–^**^2^s**^–^**^1^ for 16 DAS (ML) and 100 μmol photons m**^–^**^2^s**^–^**^1^ for 18 DAS (LL), until third leaf stage. Water content was determined on five leaf disks of the second leaf. Chlorophyll *a* fluorescence parameters were determined on leaf 2. Values represent mean and SD. F_*v*_/Fm: maximum photochemical quantum yield of PS II; Y_(II)_: effective photochemical quantum yield of PS II; ETR: electron transport rate in μmol electron m**^–^**^2^s**^–^**^1^. Letters represent significant differences between light intensities for ANOVA with Tukey’s *post hoc* test *P* < 0.05, *n* = 6.*

**TABLE 2:** Photosynthesis, stomatal conductance, substomatal concentration of CO_2_, transpiration and water use efficiency of cv. Propino plants grown under different light intensities.

	A	*g*_s_	C_i_	E	WUE
HL	21734 ± 1466 b	0.005 ± 0.003 a	10 ± 1 a	0.045 ± 0.013 a	55 ± 13 b
ML	16964 ± 2271 a	0.005 ± 0.001 a	13 ± 3 a	0.058 ± 0.010 a	58 ± 10 b
LL	16223 ± 2230 a	0.009 ± 0.002 b	36 ± 5 b	0.130 ± 0.023 b	32 ± 4 a

*Plants were grown in a 16 h:8 h light:dark photoperiod 22°C:18°C day:night under 500 μmol photons m**^–^**^2^s**^–^**^1^ (HL) for 14 DAS, 300 μmol photons m**^–^**^2^s**^–^**^1^ (ML) for 16 DAS, and 100 μmol photons m**^–^**^2^s**^–^**^1^ (LL) for 18 DAS, until third leaf stage. Values represent mean and SD from measurements taken from second leaf. A: net photosynthesis, μmol g**^–^**^1^day**^–^**^1^; *g*_*s*_: stomatal conductance, mol H_2_O g**^–^**^1^ FW day**^–^**^1^; C_*i*_: substomatal concentration of CO_2_; E: transpiration, mol⋅g**^–^**^1^⋅day**^–^**^1^; WUE: water use efficiency μmol CO_2_ mol**^–^**^1^ H_2_O. Letters represent significant differences between light intensities for ANOVA with Tukey’s *post hoc* test *P* < 0.05, *n* = 6.*

For all light treatments, the protein content was stable or varied moderately during the day in all tissues and was notably higher in blades and in the third sheath compared to the sheaths of leaves 1 and 2 (Supplementary Figure 3). Light affected the protein content of the blades and sheaths of first and second leaves, with higher contents for the HL treatment, but the protein content of the actively growing third leaf was not affected by light intensity. At the whole shoot level, the total content of proteins was decreased by up to 20% for plants grown under LL and ML compared to HL plants.

### Metabolite Levels Change Qualitatively and Quantitatively in Response to Light Intensity, as Well as Their Distribution Between Shoot Tissues

**FIGURE 1:**
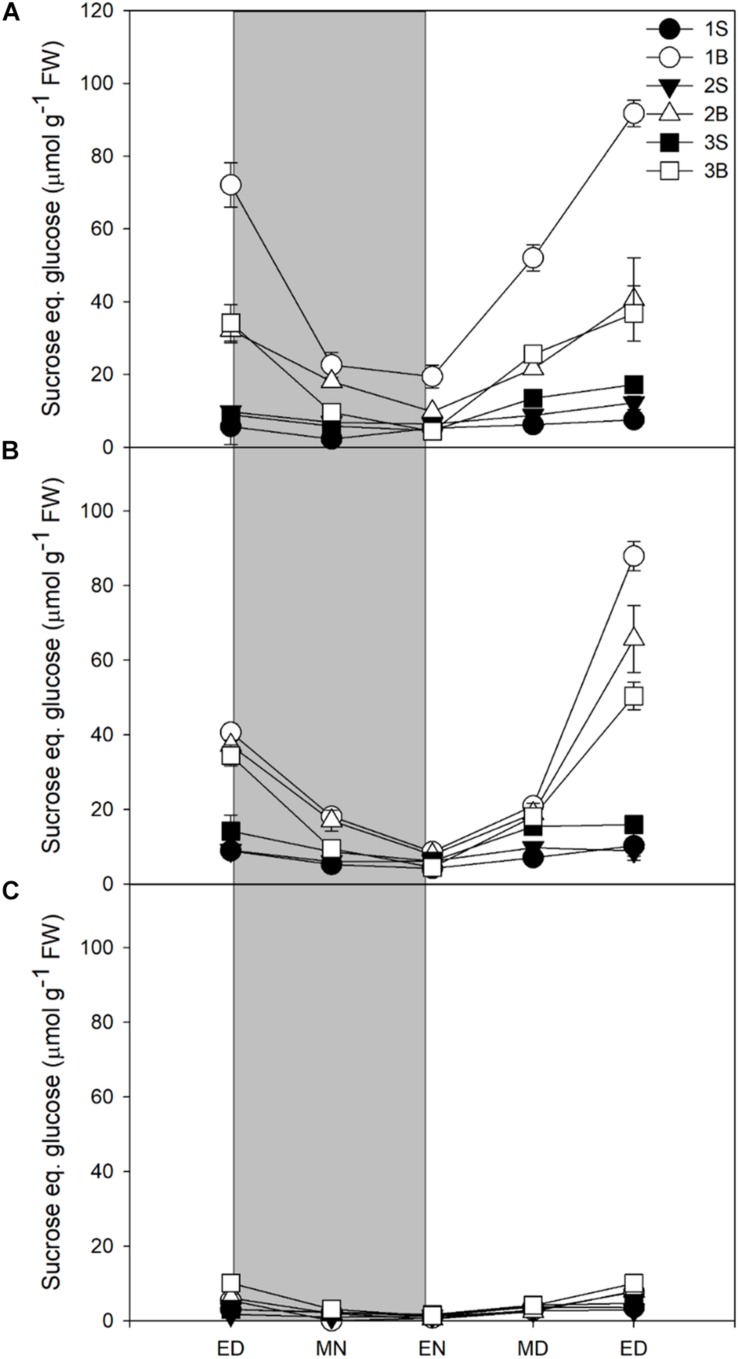
Diurnal sucrose levels of barley cv. Propino plants grown under three light intensities. Sucrose levels of plants grown for **(A)** 14 DAS under 500 μmol photons m^–2^s^–1^ (HL), **(B)** 16 DAS under 300 μmol photons m^–2^s^–1^ (ML) and **(C)** 18 DAS under 100 μmol photons m^–2^s^–1^ (LL), until third leaf stage. Plants were grown in a 16 h:8 h light:dark photoperiod 22°C:18°C day:night. 1S: 1st leaf sheath; 1B: 1st leaf blade; 2S: 2nd leaf sheath; 2B: 2nd leaf blade; 3S: 3rd leaf sheath; 3B: 3rd leaf blade; ED: end of day; MN: middle of night; EN: end of night; MD: middle of day; ED2: end of subsequent day; FW: fresh weight; gray panels: night period; error bar represents SD; *n* = 3. Statistical analyses were performed to assess differences between time points, tissues and light treatments by ANOVA with Tukey’s *post hoc* test *P* < 0.05, and results are available in Supplementary Table 1.

Glucose levels did not vary in response to light intensity and highest levels were found in younger sheaths, with very low levels in blades (Supplementary Figure 4). The sheaths showed a decrease in glucose levels at night, but without complete degradation of the glucose pool. Fructose followed a similar pattern to glucose, accumulating mainly in sheaths and presenting an incomplete consumption at end of night, but it was accumulated in smaller quantities than glucose (Supplementary Figure 5). Sucrose content was strongly affected by light intensity and mostly accumulated in blades. Sucrose represented ca 30% of reserves at end of day in plants grown under HL and ML, but only 11% in plants under LL (Table 3). Plants grown under HL showed the highest content of sucrose, especially in the first blade (Figure 1A), whilst the plants grown under LL showed very little accumulation of sucrose, the highest levels being observed in the youngest blade Figure 1). For all light treatments, the sucrose accumulated at ED was largely consumed by the end of the night, with 70, 76, and 82% of sucrose consumed for plants grown under HL, ML, and LL (Figure 1 and Table 3).

**TABLE 3:** Accumulation and mobilization of C reserves in cv. Propino shoots.

	HL	ML	LL
**Total accumulation at end of day (C μmol g^–1^ FW)**			
Glucose	28 ± 5 a	27 ± 1 a	21 ± 2 a
Fructose	6 ± 1 a	7 ± 1 a	5 ± 0 a
Sucrose	225 ± 10 c	184 ± 3 b	29 ± 3 a
Starch	111 ± 5 c	69 ± 1 b	37 ± 3 a
Fructans	113 ± 9 b	32 ± 3 a	31 ± 4 a
Malate	258 ± 10 c	230 ± 6 b	128 ± 5 a
Total^1^	741 ± 21 c	548 ± 4 b	251 ± 4 a
**Contribution of metabolites to the total C accumulated at end of day (%)**			
Glucose	4	5	8
Fructose	1	1	2
Sucrose	30	34	12
Starch	15	13	15
Fructans	15	6	12
Malate	35	42	51
**Turnover of metabolites (% metabolised at night)**			
Glucose	59	71	53
Fructose	53	65	42
Sucrose	70	76	82
Starch	73	83	76
Fructans	63	51	55
Malate	47	42	43
Total^2^	61	61	55
**Contribution of individual metabolites to the total C consumed at night (%)**			
Glucose	4	6	8
Fructose	1	1	2
Sucrose	35	42	18
Starch	18	17	20
Fructans	16	5	12
Malate	27	29	40

*Plants were grown in a 16 h:8 h light:dark photoperiod at 22°C:18°C day:night with 500 μmol photons m^–2^s^–1^. (HL) 300 μmol photons m^–2^s^–1^ (ML), 100 μmol photons m^–2^s^–1^ (LL). FW: fresh weight. Letters represent differences between treatments by ANOVA with Tukey’s *post hoc* test *P* < 0.05; *n* = 3. ^1^Total of reserves accumulated at end of day in the shoot; ^2^Percentage of the total reserves consumed at night from total reserves accumulated at end of day.*

**FIGURE 2:**
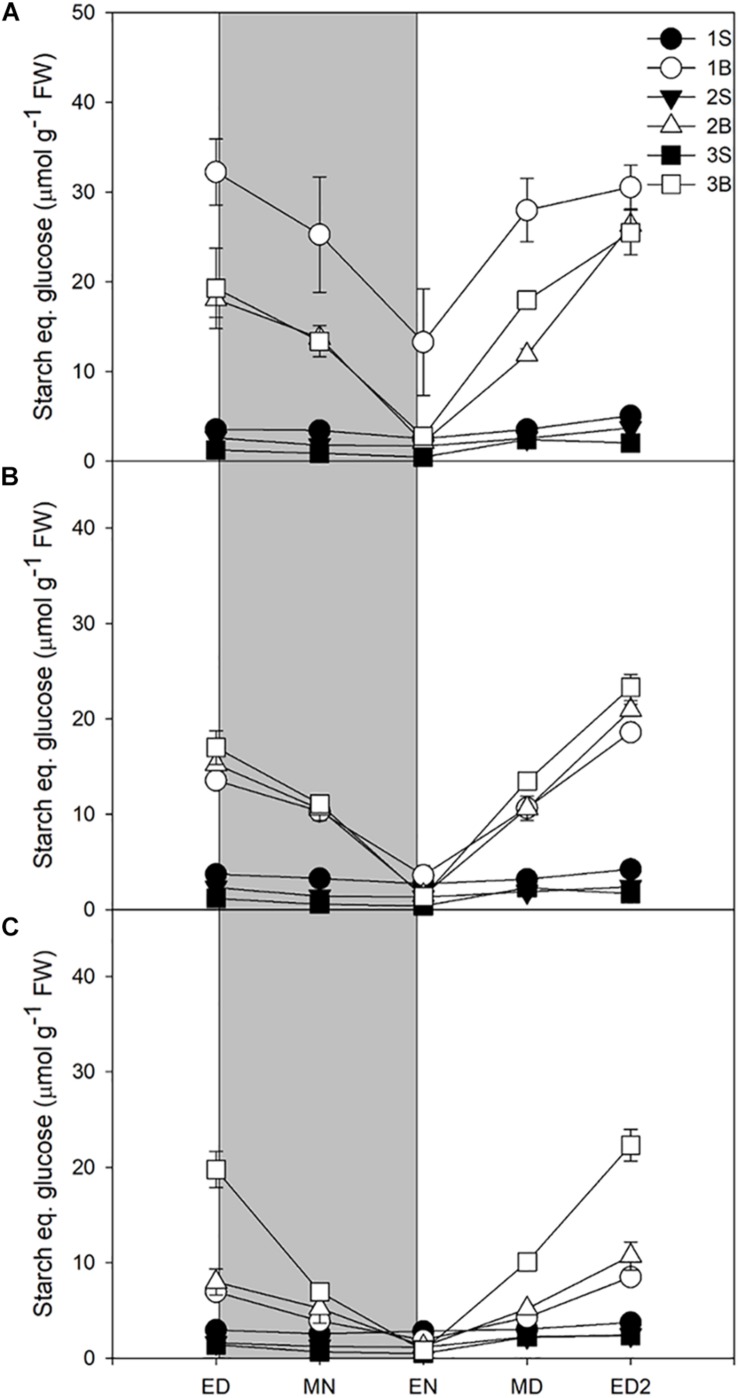
Diurnal starch levels of barley cv. Propino plants grown under three light intensities. Starch levels of plants grown for **(A)** 14 DAS under 500 μmol photons m^–2^s^–1^ (HL), **(B)** 16 DAS under 300 μmol photons m^–2^s^–1^ (ML) and **(C)** 18 DAS under 100 μmol photons m^–2^s^–1^ (LL), until third leaf stage. Plants were grown in a 16 h:8 h light:dark photoperiod 22°C:18°C day:night. 1S: 1**st** leaf sheath; 1B: 1**st** leaf blade; 2S: 2**nd** leaf sheath; 2B: 2nd leaf blade; 3S: 3rd leaf sheath; 3B: 3**rd** leaf blade; ED: end of day; MN: middle of night; EN: end of night; MD: middle of day; ED2: end of subsequent day; FW: fresh weight; gray panels: night period; error bar represents SD; *n* = 3. Statistical analyses were performed to assess differences between time points, tissues and light treatments by ANOVA with Tukey’s post hoc test *P* < 0.05, and results are available in Supplementary Table 1.

Starch accumulation increased moderately in response to increased light intensities, represented only ca 14% of the metabolites accumulated at ED, and was largely consumed at night, with a turnover of 73–83% (Table 3). Its accumulation during the daytime and consumption at night resembled the patterns observed for sucrose, at the exception of the third blade where starch levels and turnover were identical for the three light treatments Figure 2).

**FIGURE 3:**
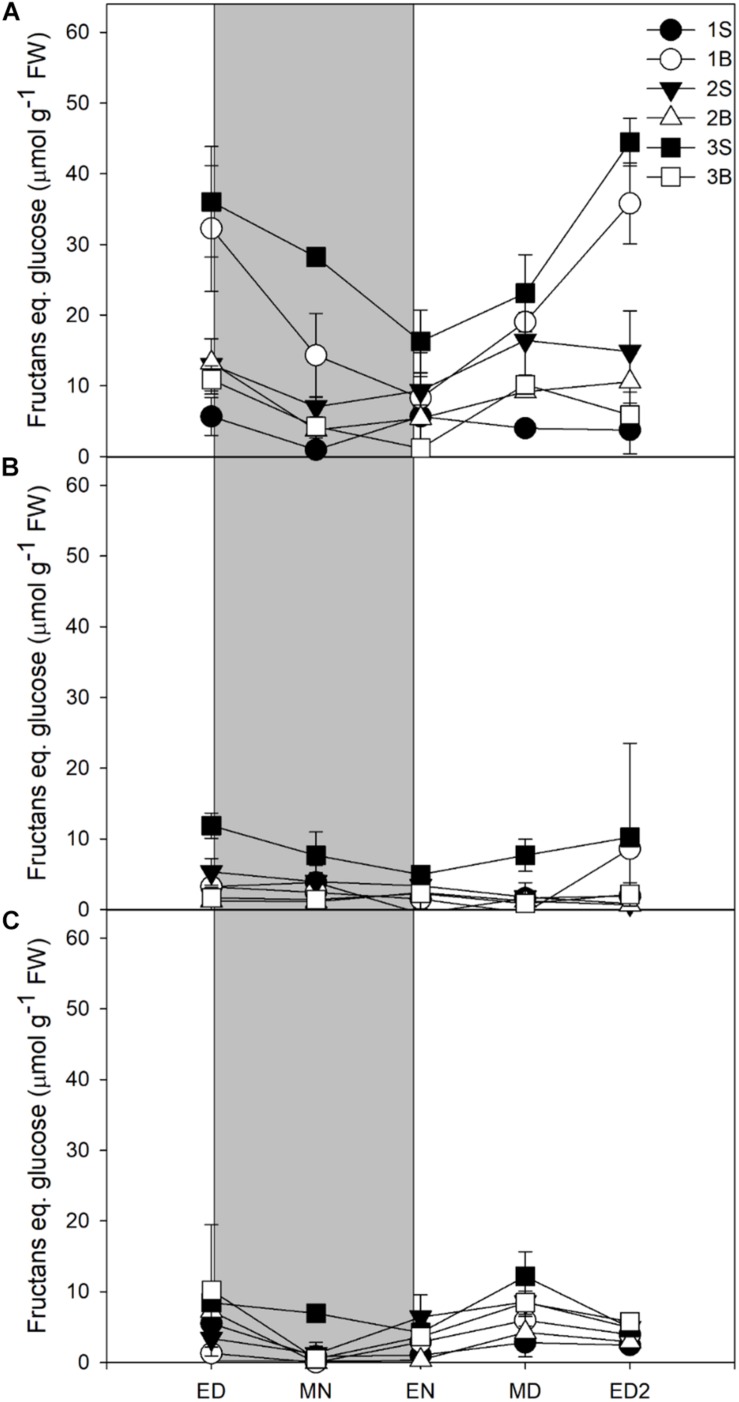
Diurnal fructans levels of barley cv. Propino plants grown under three light intensities. Fructans levels of plants grown for **(A)** 14 DAS under 500 μmol photons m**^–^**^2^s**^–^**^1^ (HL), **(B)** 16 DAS under 300 μmol photons m**^–^**^2^s**^–^**^1^ (ML) and **(C)** 18 DAS under 100 μmol photons m**^–^**^2^s**^–^**^1^ (LL), until third leaf stage. Plants were grown in a 16 h:8 h light:dark photoperiod 22°C:18°C day:night. 1S: 1st leaf sheath; 1B: 1st leaf blade; 2S: 2nd leaf sheath; 2B: 2nd leaf blade; 3S: 3rd leaf sheath; 3B: 3rd leaf blade; ED: end of day; MN: middle of night; ED: end of night; MD: middle of day; ED2: end of subsequent day; FW: fresh weight; gray panels: night period; error bar represents SD; *n* = 3. Statistical analyses were performed to assess differences between time points, tissues and light treatments by ANOVA with Tukey’s *post hoc* test *P* < 0.05, and results are available in Supplementary Table 1.

Fructans accumulation was mostly observed in plants grown under HL, the plants grown under ML and LL accumulating four times less (Figure 3). Fructans accumulated in both blades and sheaths, with the first blade (older blade) and the third sheath (younger and developing tissue) of plants grown under HL showing the highest accumulation of fructans at ED. In contrast to sucrose and starch, the fructans were not fully consumed at EN (Figure 3A).

**FIGURE 4:**
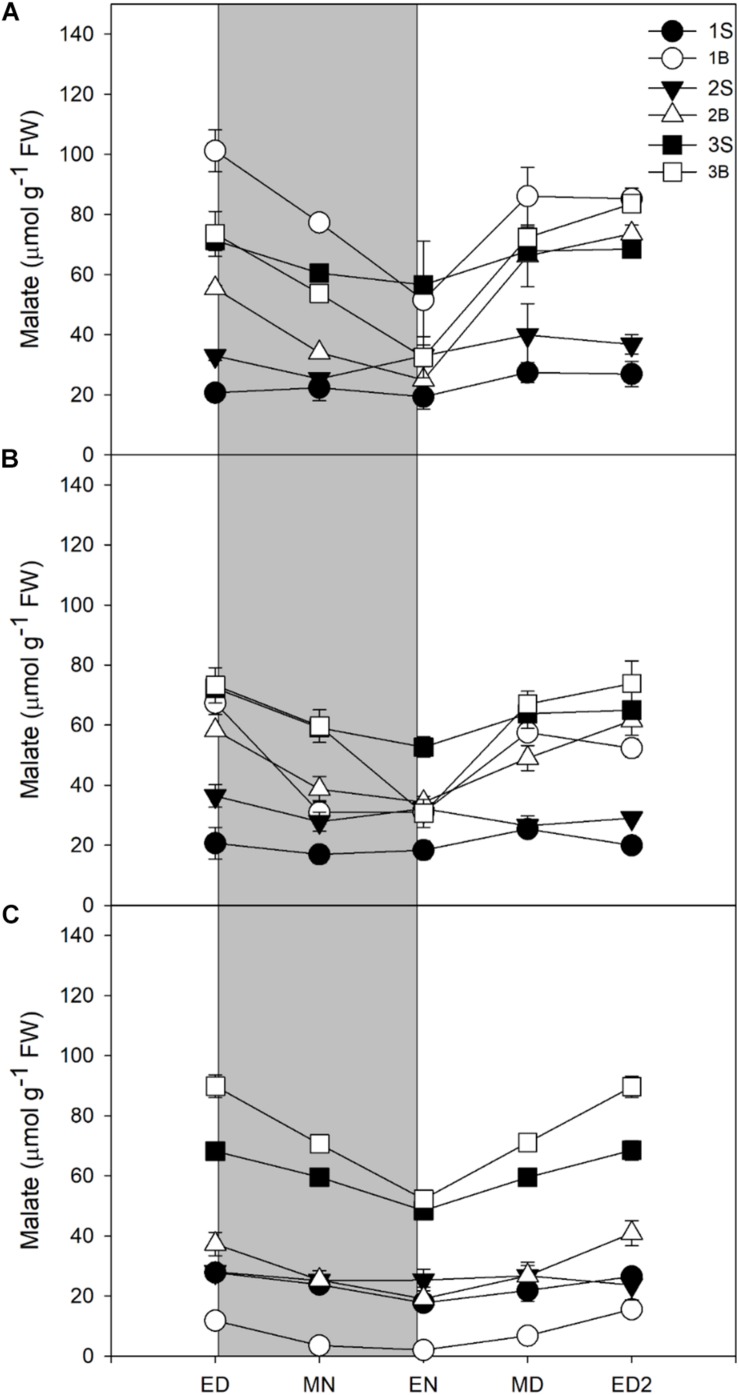
Diurnal malate levels of barley cv. Propino plants grown under three light intensities. Malate levels of plants grown for **(A)** 14 DAS under 500 μmol photons m**^–^**^2^s**^–^**^1^ (HL), **(B)** 16 DAS under 300 μmol photons m**^–^**^2^s**^–^**^1^ (ML) and **(C)** 18 DAS under 100 μmol photons m**^–^**^2^s**^–^**^1^ (LL), until third leaf stage. Plants were grown in a 16 h:8 h light:dark photoperiod 22°C:18°C day:night. 1S: 1st leaf sheath; 1B: 1st leaf blade; 2S: 2nd leaf sheath; 2B: 2nd leaf blade; 3S: 3rd leaf sheath; 3B: 3rd leaf blade; ED: end of day; MN: middle of night; EN: end of night; MD: middle of day; ED2: end of subsequent day; FW: fresh weight; gray panels: night period; error bar represents SD; *n* = 3. All statistics are available in Supplementary Table 1.

Unexpectedly, the malate levels in barley leaf tissues were very high, with malate being quantitatively the main soluble C compound accumulated by barley leaves in all three light treatments. Although malate content was reduced with a decrease in light intensity (Figure 4 and Table 3), it represented 34% (HL), 41% (ML), and 50% (LL) of the reserves accumulated at the end of the day, so its importance increased qualitatively (Table 3).

In all light conditions, malate levels were higher in blades than sheaths, and it was only partially consumed at night, leading to high levels of malate remaining at EN. Only 42–47% of the amounts accumulated were consumed at night, in contrast to 70–83% for starch and sucrose, or 51–63% for fructans (Table 3). Despite a low consumption of malate pool during the night, malate was either the second (HL and ML) or the main C contributor to night consumption (LL, Table 3).

For all light treatments, malate levels were higher in the third sheath compared to the sheaths of first and second leaves. Malate levels in third sheaths did not varied amongst the treatments while in the first and second blade it showed a reduction in its levels when light intensity decreased (Figure 4). The youngest blade – third blade – showed no difference in malate levels between HL and ML but increased in the third blade of plants grown under LL, in stark contrast with other metabolites where their levels increased with increased light intensities.

**FIGURE 5:**
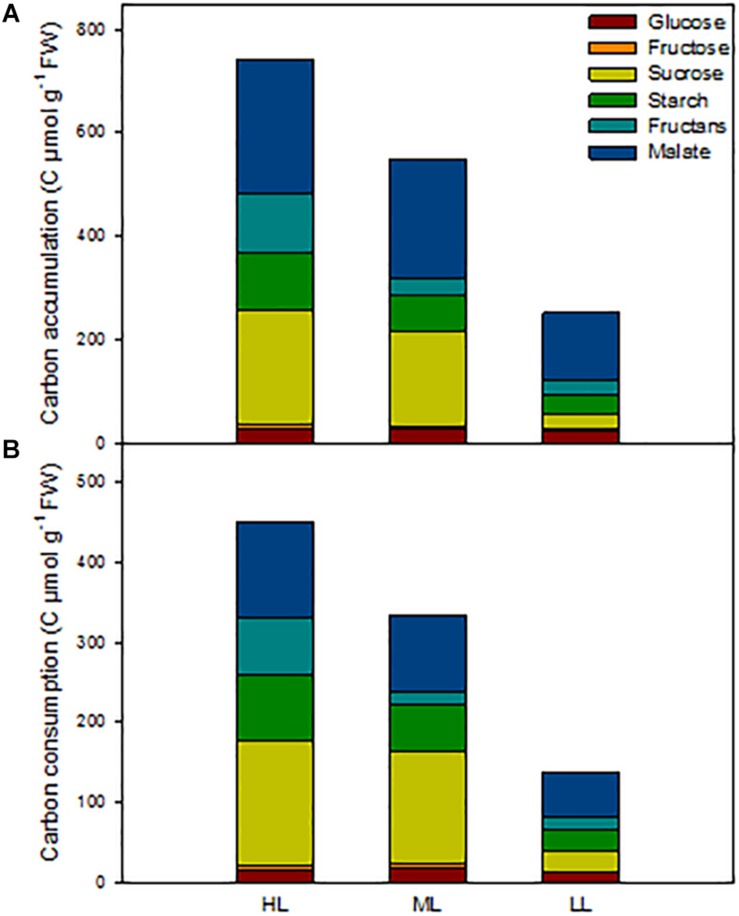
Estimation of non-structural carbon reserves in barley cv. Propino shoots at end of the day of plants grown under three light intensities. **(A)** Total C amount at end of the day in whole shoots; **(B)** total C amount consumed during the night. Plants were grown in a 16 h:8 h light:dark photoperiod at 22°C:18°C day:night for 14 DAS under 500 μmol photons m^–2^s^–1^ (HL), 16 DAS under 300 μmol photons m^–2^s^–1^ (ML) and 18 DAS under 100 μmol photons m^–2^s^–1^ (LL), until third leaf stage. FW: fresh weight; *n* = 3. Statistical analyses were performed to assess differences between time points, tissues and light treatments by ANOVA with Tukey’s post hoc test *P* < 0.05, and results are available in Supplementary Table 1.

In order to get an overview of the C status of the barley plants, we calculated the total amount of carbon present in the metabolites analyzed at ED in shoots, as well as the amount of C consumed during the night (Figure 5 and Table 3). We also investigated in which tissues metabolites accumulated and which tissues contributed to growth and cell maintenance at night (Supplementary Figures 6, 7). The carbon status at ED was significantly reduced with decrease in light intensity, being 25% less for plants under ML and 65% less in plans under LL compared to HL (Figure 5 and Table 3). When investigating the location of the reserves (Supplementary Figure 6), it appeared that under HL the mature blade of leaf 1 accumulated large amounts of metabolites compared to plants grown under ML. The first blade – older blade – of plants grown under HL contained mainly sucrose and malate, while the leaf 1 sheath contained the least amounts of metabolites (Supplementary Figure 6A). Second and third leaves accumulated similar total C content in both sheaths and blades, but sheaths contained mainly malate and fructans, while blades accumulated more sucrose, malate and starch (Supplementary Figure 6A). Plants grown under ML presented similar amounts of metabolites in their blades, and sheaths slightly less at the exception of the youngest sheath (Supplementary Figure 6B). Under low light, leaves 1 and 2 contained low amounts of metabolites, mostly malate, while the actively growing third leaf contained similar levels of C to those observed under ML and HL, the main metabolite being malate (Supplementary Figure 6C).

The patterns of C consumption at night per each individual tissue resembled the patterns observed for accumulation (Supplementary Figure 7). At whole plant level, the total consumption of carbon at night time was reduced with decreased light intensity Figure 5) and the percentage of metabolites used at night was similar between light conditions, with 61% for both HL and ML and 55% for LL (Table 3). Although plants grown under HL and ML showed similar consumption levels of metabolites at night, the contribution of each metabolite was different. Plants under HL consumed mostly sucrose and malate, but plants grown under ML consumed more sucrose, while plants grown under LL consumed mostly malate and then starch and sucrose (Figure 5 and Table 3).

## Discussion

### Light Intensity Affects Barley Plant Development, CO2 Assimilation and Protein Content

The development of the spring barley cv. Propino was very dependent on light intensity, the plants grown under HL growing the fastest. The establishment of the first leaf was slowed by the low light intensity (not shown), but when third leaf appeared, the elongation rates of second and third leaves were similar for the three light intensities (Supplementary Figure 2). However, CO_2_ assimilation in plants grown under ML and LL was reduced by about 21% compared to HL plants (Table 2). It is likely explained by a lower dry weight content of the leaves of LL plants, with 8% compared to 12 and 10% for HL and ML plants, respectively (Table 1). Interestingly, plants grown under LL also showed a decrease of 20% in their total amount of proteins (Supplementary Figure 3). Blenkinsop and Dale (1974), reported a reduction of 70% of the protein content, a decrease in chlorophyll *a* content and photosynthesis when a leaf was totally shaded.

### Light Intensity Affects the Total Amount and Composition of Metabolite Leaf Pools

Changes in light intensity not only impacted the total amount of photoassimilates accumulated during daytime, but also their composition and location. Malate was the main metabolite present in barley leaves, representing 34–50% of the total amount of photoassimilates. This organic acid had never been identified as a major metabolite in C_3_ photosynthesis species and sucrose was considered the main photoassimilate in barley, with smaller amounts of starch, hexoses and fructans (Gordon et al., 1977, 1979; Gordon et al., 1980; Farrar and Farrar, 1985). Malate contribution to the pool of photoassimilates decreased with higher light intensities while sucrose showed the opposite. Malate was the main metabolite contributing to C use at night under LL and the second under HL and ML (Table 3). Malate is an intermediate of the TCA cycle and major component of the malate valve. It has many roles in plant metabolism as precursor of other metabolites, regulation of redox potential, root exudation and stomata movement (Fernie and Martinoia, 2009). Here, we propose that in barley it plays a role as a major alternative source of carbon to night growth and maintenance, in particular under light limitation. A role in the control of turgor is also likely, because its levels remaining very high at end of night (Figure 4 and Table 3).

### Light Intensity Affects the Spatial Distribution of Metabolites

Interestingly, light intensity not only affected the composition of the transient C stores, but also had a strong impact on their location, with the highest levels observed in old blades when plants were grown under HL whilst plants grown under LL preferentially accumulated C metabolites in youngest leaves (Supplementary Figure 6). However, the youngest leaf accumulated very similar metabolite levels for all three light intensities. We propose that barley is preferentially storing its resources in the youngest actively growing tissue where they are required for growth, and then store the excess in other tissues. Under shade by neighboring plants, Arabidopsis actively reallocates starch to the hypocotyls, likely to provide the C required for elongation (de Wit et al., 2018). Under LL, barley not only allocates starch or sucrose, but also malate to the youngest leaf. This preferential accumulation of C in barley to young tissues also occurs under continuous light, with reducing sugars and sucrose accumulating in the terminal meristem and roots, while starch is retained in the leaf (Gordon et al., 1979). In Arabidopsis, the translocation of C resources has been described to also vary during the diurnal cycle (Kolling et al., 2015), the export of photoassimilates toward the sink tissues being particularly active in the morning. However, we did not find any evidence for a more active transport of photoassimilates at certain times of the day. It might be explained by the differential diurnal growth pattern of Arabidopsis compared to monocots. Arabidopsis grows mostly in the morning while barley shows very stable growth rates during the day (Poire et al., 2010).

### Light Intensity Does Not Affect the Percentage of Metabolites Consumed at Night

For the three growth conditions, around 55–61% of the total C accumulated at ED was consumed during the night and interestingly, each metabolite was similarly consumed for the three light intensities, with malate showing the lowest turnover (42–47%) and starch the highest (73–83%; Table 3). However, the contribution of each leaf to the overall C consumed during the night varied a lot between the three growth conditions (Supplementary Figures 6, 7). Thus, light intensity does affect the spatial allocation of resources, the type of metabolites accumulated, but the turnover of these metabolites is very similar for all light intensities, suggesting a tight regulation.

## Conclusion

The development of barley is dependent on light intensity. In response to decreases in light intensities, we observed an adjustment in the metabolism of transient reserves with a decrease in sucrose, starch and malate accumulation. However, this decrease was not equivalent for all these three major carbon pools, sucrose and to some extent starch levels being drastically reduced by a decrease in light intensity while malate levels remained high even under low light. Thus, the pools of metabolites are differentially affected by variation in CO_2_ assimilation, suggesting a rewiring of the metabolic pathways. Moreover, changes in light intensities also affected the spatial distribution of the C pools, low light grown plants showing the highest amounts of metabolites in their youngest tissues. The next steps will be to document if other environmental factors do affect the partitioning of photosynthetates between starch, sucrose and malate, as well as their mobilization at night time, and identify the genes involved. The identification of the genes could be achieved via Genome Wide Association Mapping. It would then allow to document to which extent our observations made on a spring barley cultivar can be extended to other barley varieties.

## Data Availability Statement

All datasets generated for this study are included in the article/Supplementary Material.

## Author Contributions

KB and RS designed the experiments and wrote the manuscript. KB, AE-F, HM, JF and SB performed the experiments. KB, AE-F, HM, JF, SB, and RS analyzed the data. All authors reviewed the manuscript.

## Conflict of Interest

The authors declare that the research was conducted in the absence of any commercial or financial relationships that could be construed as a potential conflict of interest.
