# Supplementary material for: Transient Carbon Reserves in Barley: Malate, Sucrose and Starch Are the Main Players, Their Quantitative Involvement Being Light Intensity Dependant
Source: Front Plant Sci. 2020 Mar 6;11:209. doi: 10.3389/fpls.2020.00209 (PMC7068212; doi:10.3389/fpls.2020.00209)
Supplement: Supplementary file 2 [file Table_2.DOCX]

**Supplementary figure 1 Height and fresh biomass of cv. Propino plants grown under three light intensities at the last harvest timepoint.** (A): height at 14 DAS under 500 µmol photons m^-2^s^-1^ (HL), at 16 DAS under 300 µmol photons m^-2^s^-1^ (ML) and at 18 DAS under 100 µmol photons m^-2^s^-1^ (LL); (B): fresh weight biomass. Plants were grown in a 16h:8h light:dark photoperiod at 22°C:18°C day:night. DAS: days after sowing; error bar represents SD; n=6; letters represent significant differences (ANOVA with Tukey post hoc test P<0.05).

**Supplementary figure 2- Elongation rates of second and third leaves of cv. Propino plants grown under three light intensities**. (A): elongation rate of second leaves. (B): elongation rate of third leaves. Plants were grown in a 16h:8h light:dark photoperiod for 14 DAS under 500 µmol photons m^-2^s^-1^ (HL), 16 DAS under 300 µmol photons m^-2^s^-1^ (ML) and 18 DAS under 100 µmol photons m^-2^s^-1^ (LL); error bar represents SD; n=6. No elongation rates were significantly different from others for the two leaves (ANOVA with Tukey post hoc test P<0.05).


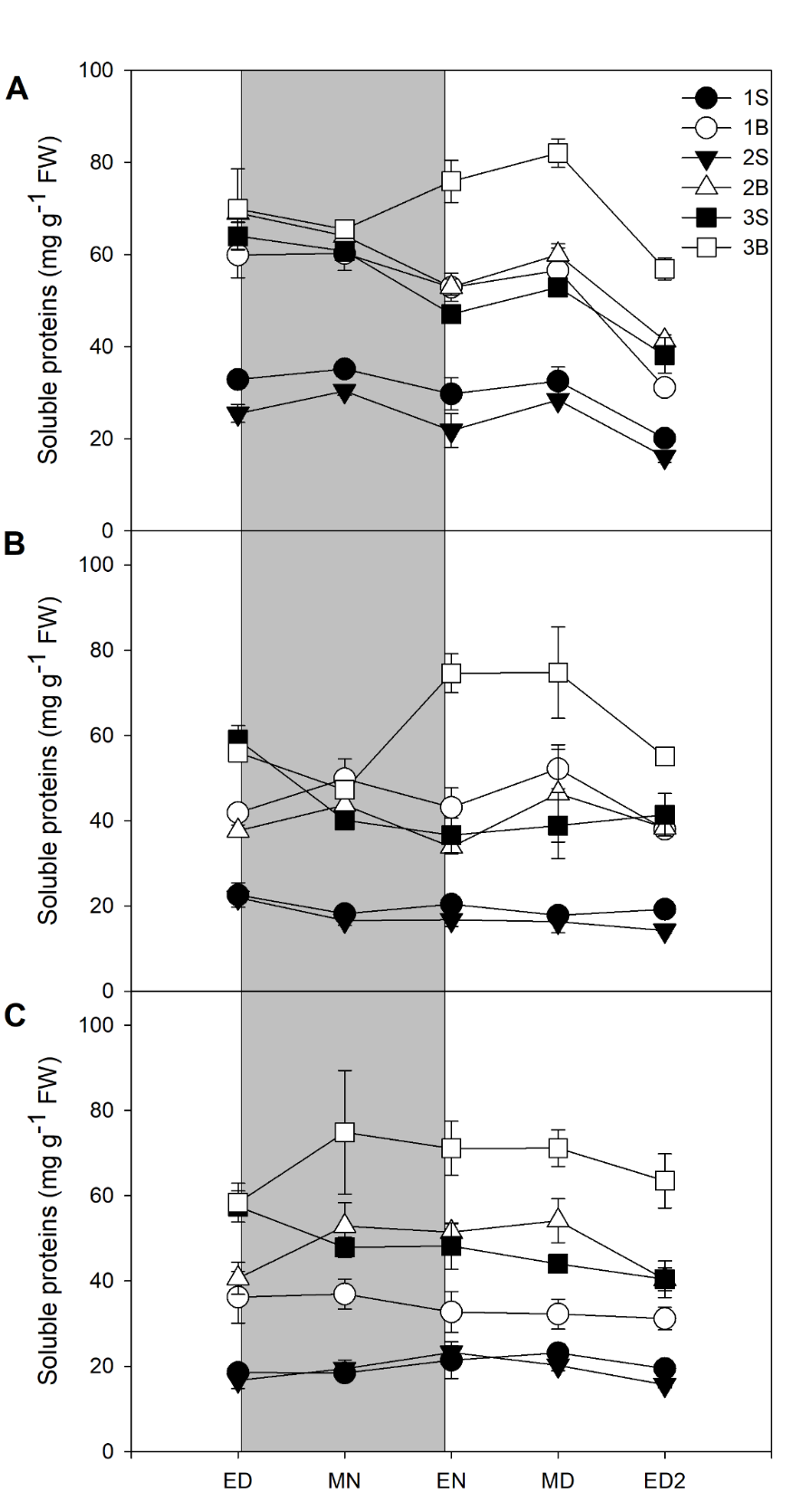


**Supplementary figure 3 Diurnal protein contents of cv. Propino plants grown under three light intensities**. Protein levels of plants grown for (A) 14 DAS under 500 µmol photons m^-2^s^-1^ (HL), (B) 16 DAS under 300 µmol photons m^-2^s^-1^ (ML) and (C) 18 DAS under 100 µmol photons m^-2^s^-1^ (LL), until third leaf stage. Plants were grown in a 16h:8h light:dark photoperiod 22°C:18°C day:night. 1S: 1^st^ leaf sheath; 1B: 1^st^ leaf blade; 2S: 2^nd^ leaf sheath; 2B: 2^nd^ leaf blade; 3S: 3^rd^ leaf sheath; 3B: 3^rd^ leaf blade; ED: end of day; MN: middle of night; EN: end of night; MD: middle of day; ED2: end of subsequent day; FW: fresh weight; grey panels: night period; error bar represents SD; n= 3. Statistical analyses were performed to assess differences between time points, tissues and light treatments by ANOVA with Tukey’s post hoc test P<0.05, and results are available in Supplemental Table 1.


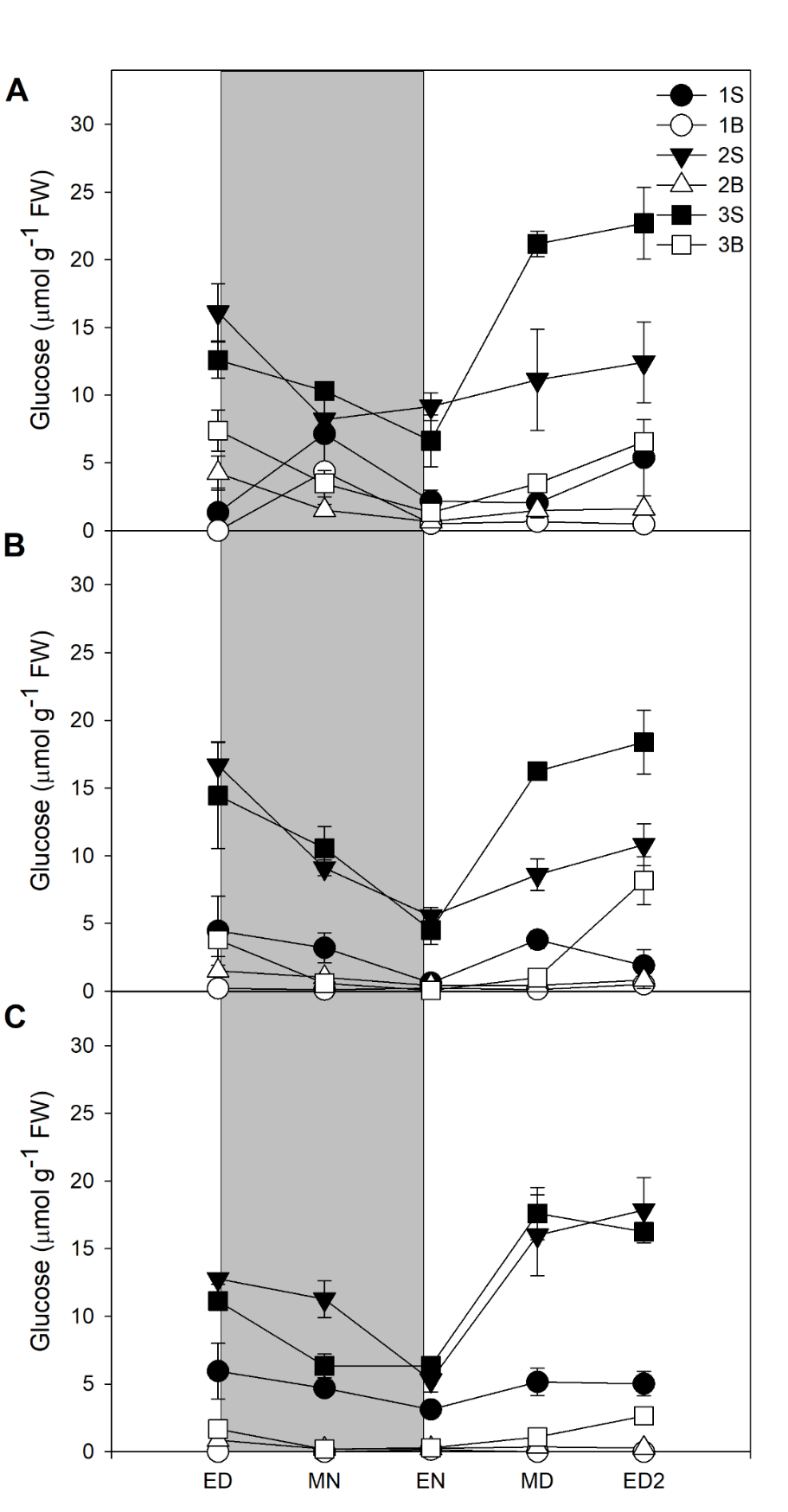


**Supplementary figure 4 Diurnal glucose levels of cv. Propino plants grown under three light intensities**. Glucose levels of plants grown for (A) 14 DAS under 500 µmol photons m^-2^s^-1^ (HL), (B) 16 DAS under 300 µmol photons m^-2^s^-1^ (ML) and (C) 18 DAS under 100 µmol photons m^-2^s^-1^ (LL), until third leaf stage. Plants were grown in a 16h:8h light:dark photoperiod 22°C:18°C day:night. 1S: 1^st^ leaf sheath; 1B: 1^st^ leaf blade; 2S: 2^nd^ leaf sheath; 2B: 2^nd^ leaf blade; 3S: 3^rd^ leaf sheath; 3B: 3^rd^ leaf blade; ED: end of day; MN: middle of night; EN: end of night; MD: middle of day; ED2: end of subsequent day; FW: fresh weight; grey panels: night period; error bar represents SD; n= 3. Statistical analyses were performed to assess differences between time points, tissues and light treatments by ANOVA with Tukey’s post hoc test P<0.05, and results are available in Supplemental Table 1.


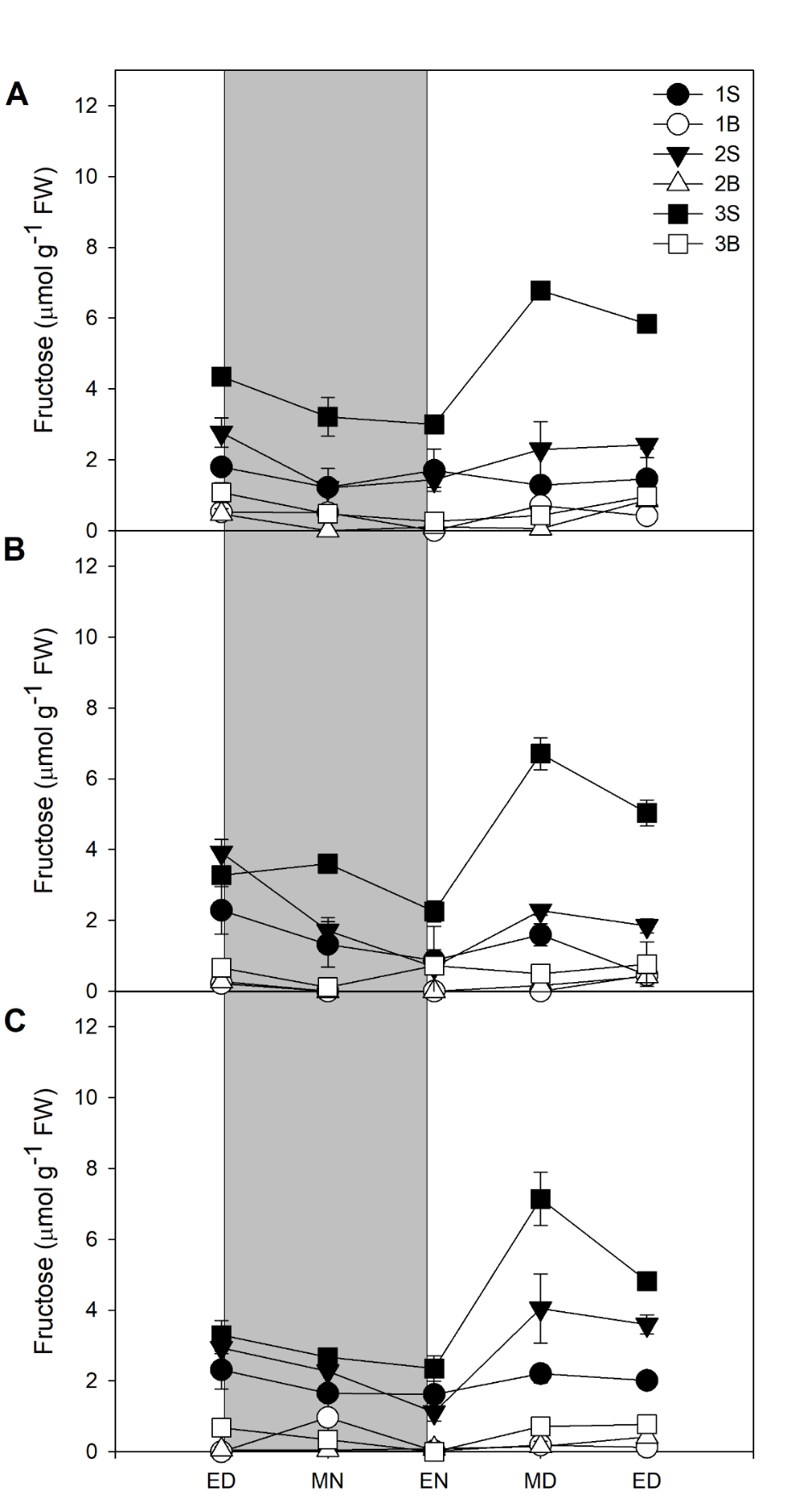


**Supplementary figure 5 Diurnal fructose levels of Propino cv. plants grown under three light intensities**. Fructose levels of plants grown for (A) 14 DAS under 500 µmol photons m^-2^s^-1^ (HL), (B) 16 DAS under 300 µmol photons m^-2^s^-1^ (ML) and (C) 18 DAS under 100 µmol photons m^-2^s^-1^ (LL), until third leaf stage. Plants were grown in a 16h:8h light:dark photoperiod 22°C:18°C day:night. 1S: 1^st^ leaf sheath; 1B: 1^st^ leaf blade; 2S: 2^nd^ leaf sheath; 2B: 2^nd^ leaf blade; 3S: 3^rd^ leaf sheath; 3B: 3^rd^ leaf blade; ED: end of day; MN: middle of night; EN: end of night; MD: middle of day; ED2: end of subsequent day; FW: fresh weight; grey panels: night period; error bar represents SD; n= 3. Statistical analyses were performed to assess differences between time points, tissues and light treatments by ANOVA with Tukey’s post hoc test P<0.05, and results are available in Supplemental Table 1.

**Supplementary figure 6 Carbon accumulation per organ in Propino cv. grown under three light intensities**. Composition of carbon accumulated at end of the day for whole shoot of plants grown for (A) 14 DAS under 500 µmol photons m^-2^s^-1^ (HL), (B) 16 DAS under 300 µmol photons m^-2^s^-1^ (ML) and (C) 18 DAS under 100 µmol photons m^-2^s^-1^ (LL), until third leaf stage. Plants were grown in a 12h:12h light:dark photoperiod with 500 µmol photons m^-2^s^-1^. 1S: 1^st^ leaf sheath; 1B: 1^st^ leaf blade; 2S: 2^nd^ leaf sheath; 2B: 2^nd^ leaf blade; 3S: 3^rd^ leaf sheath; 3B: 3^rd^ leaf blade; FW: fresh weight; n= 3. Statistical analyses were performed to assess differences between time points, tissues and light treatments by ANOVA with Tukey’s post hoc test P<0.05, and results are available in Supplemental Table 1.

**Supplementary figure 7 Carbon consumption per organ in Propino cv. grown under three light intensities**. Composition of carbon pools consumed during night time for whole shoot of plants grown for (A) 14 DAS under 500 µmol photons m^-2^s^-1^ (HL), (B) 16 DAS under 300 µmol photons m^-2^s^-1^ (ML) and (C) 18 DAS under 100 µmol photons m^-2^s^-1^ (LL), until third leaf stage. Plants were grown in a 12h:12h light:dark photoperiod with 500 µmol photons m^-2^s^-1^. 1S: 1^st^ leaf sheath; 1B: 1^st^ leaf blade; 2S: 2^nd^ leaf sheath; 2B: 2^nd^ leaf blade; 3S: 3^rd^ leaf sheath; 3B: 3^rd^ leaf blade; DW: dry weight; n= 3.
